# Dynamic Associations Between Intrinsic Capacity and Disability Before Death Among Older Adults

**DOI:** 10.1093/geroni/igaf122.3920

**Published:** 2025-12-31

**Authors:** Olivia Malkowski, Kendra Plourde, Max Western, Thomas Gill

**Affiliations:** Yale University, New Haven, Connecticut, United States; Yale School of Public Health, New Haven, Connecticut, United States; University of Bath, Bath, England, United Kingdom; Yale University, New Haven, Connecticut, United States

## Abstract

Intrinsic capacity is negatively associated with disability; however, little is known about how the magnitude of this association varies leading up to death. Using time-varying effect modeling (TVEM), a statistical tool for capturing dynamic patterns of change, we evaluated the association between intrinsic capacity and disability as a function of time before death. Data were analyzed from the 746 (of 754 enrolled) participants aged 70+ years of the Precipitating Events Project cohort study who died through November 2024. Intrinsic capacity was assessed at baseline and subsequently at 18-month intervals using 25 self-reported and physical performance measures. Intrinsic capacity scores (range: 0–100) were derived using Bayesian Multilevel Item Response Theory. Disability was assessed monthly and defined as dependence in instrumental activities of daily living. TVEM was applied to evaluate the association between intrinsic capacity and disability, anchored to participants’ time of death. Among the 746 decedents (64.2% female), the mean age at death was 88.4 years. Trajectories using a backwards timescale spanning 20 years showed a gradual decline in average intrinsic capacity from 80 to 47 points; the change in disability prevalence was non-linear. The inverse association between intrinsic capacity and disability strengthened as individuals approached death. In the last four years of life, a ten-point increase in intrinsic capacity was associated with 47–48% lower odds of disability. The heightened association between intrinsic capacity and disability at the end of life underscores the importance of monitoring changes in a person’s intrinsic capacity, which might offer opportunities for preventive action.

